# Refugee Employment Integration Heterogeneity in Sweden: Evidence From a Cohort Analysis

**DOI:** 10.3389/fsoc.2020.00044

**Published:** 2020-07-02

**Authors:** Pieter Bevelander, Marc-André Luik

**Affiliations:** Malmö Institute for Studies of Migration, Diversity and Welfare, Malmö University, Malmö, Sweden

**Keywords:** refugees, employment, cohort, Sweden, integration

## Abstract

Sweden, like many other European countries, has lower employment levels for the foreign-born compared to native-born Swedes. To some extent, this could be due to the country's relatively large intake of refugees. However, few studies have focused entirely on the employment integration of these refugees. In order to fill this gap, we use detailed longitudinal Swedish register data of three arrival cohorts (1998–2000). These data cover the employment of refugees from different countries of origin in Sweden in the first 12 years since their arrival. In line with related work and theoretical considerations and with respect to group characteristics, outmigration, and employment integration over time, we find differences between dissimilar groups of refugees. The findings concerning employment integration decrease to a small degree after rich regression adjustments. Moreover, maybe more surprisingly, we find a very similar result within the main groups of refugees from countries such as Bosnia, Ethiopia, and Eritrea. Women from these groups, in particular, have similar or higher employment probabilities than Swedish-born women after between 5 and 8 years in the country. Overall, each group managed to catch up to a non-negligible, yet varying, degree compared to related empirical evidence from other countries. The role of contextual factors in the refugee sending and receiving countries is highlighted.

## Introduction

The number of asylum-seekers entering Europe rose dramatically following the Arab Spring in 2011 and especially during late 2015; consequently the integration of refugees in new labor markets has been high on the political agenda. Notwithstanding the high increase during 2015, Sweden is one of the few countries in Europe to have admitted a large refugee population over recent decades. Besides, Sweden is also a country with highly ambitious labor-market integration policies (see MIPEX 2015: http://www.mipex.eu/) but which has, at the same time, a considerable native–immigrant employment gap compared to other OECD countries (OECD, 2017)^1^.

One reason for the employment gap could be the relatively large intake of refugees who, according to a number of studies, show lower employment levels compared to labor and family-reunion migrants. In other words, controlling for several demographic and human-capital characteristics reveals that there remain differences in the probability of obtaining employment between refugees and other immigration categories (Bevelander, 2011; Bakkaer, 2015; Dustmann et al., 2017). Refugees are less favorably selected according to labor-market skills and should therefore have a longer period of adaption to host-country labor markets (Borjas, 1987; Chiswick, 2008). Traumatic experiences and long and uncertain asylum procedures inducing insecurity and affecting mental health in a negative way can also be prejudicial to their obtaining employment (Bakker et al., 2013; Hainmueller et al., 2016; Dustmann et al., 2017).

Although the unpacking of the heterogeneous group of immigrants by entrance category is a step in the right direction in understanding the variation in the native–immigrant employment gap between countries, the immigrant sub-category of refugees is also largely dissimilar. Refugees to Sweden have arrived over different periods, for diverse reasons, from different parts of the world and possess various characteristics, skills, and traits. The relevance and the contribution of this study lies in the fact that, by using high-quality individual register data on admission status and country of birth, we can contrast refugee employment integration patterns from different parts of the world. Moreover, the register information that we use on the relatively high intake of refugees in Sweden also allows us to follow individuals in a longitudinal framework over time and study the refugee cohort arriving between 1998 and 2000 in detail, both from a dynamic perspective as well as by mitigating outmigration bias caused by return migration—which is difficult to deal with in cross-sectional data^2^.

The research questions we are focusing on are:

1. To what extent does employment integration vary between male and female refugees by country of origin?
2. Do we observe, over time, a “catching up” or a “falling behind” for the different groups compared to native employment levels?
3. Can employment integration heterogeneity by country be explained by differences in observable characteristics such as demographics and levels of human capital?

The reminder of this paper is as follows. In the next section we provide the context for the study, followed by a section detailing earlier research. We continue with a data and method section as well as providing descriptive results. After this, we show our multivariate analysis and finish by discussing the results in the conclusion.

## The Swedish Context

At the end of 2018, about 19% of Sweden's population was born abroad, making it one of the top countries in the European Union for the reception of immigrants, only surpassed by Switzerland and Luxembourg. Although diversified in their reasons for entering Sweden, a significant proportion of the immigration to Sweden over the last 40 years has consisted of individuals seeking asylum who have subsequently gained residence. Since the early 1980s, refugees and tied movers have dominated the migration inflow, coming primarily from Eastern Europe and non-European parts of the world (Bevelander, 2011). Starting in the 1980s, the lion's share of refugee immigration came from Ethiopia, Eritrea, Iran, and other Middle Eastern countries. Individuals from Iraq and the former Yugoslavia dominated in the 1990s. Since the beginning of the new millennium, Iraqi, Somali, Syrian, and Afghan refugees have represented the largest share of the refugee intake to Sweden. Relatively liberal asylum rules have been one of the explanations for the comparatively high number of people seeking asylum in Sweden.

Swedish refugee policy is based on the UN Geneva Convention of 1951 (which Sweden signed in 1954) and established in the Swedish Aliens Act of 1989. According to this act (which has been considerably amended and reinterpreted), Sweden may give asylum to one category of refugees only, so-called *convention refugees*. These are individuals who are either stateless or are living outside the country of their nationality or former habitual residence, and who have a well-grounded fear of persecution in that country due to their race, nationality, membership of a particular social group, religious beliefs, or political opinions. These refugees have entered Sweden individually, applied for asylum and subsequently obtained a residence permit. Outside this act, Sweden obviously cooperates with the UN High Commissioner for Refugees, the UNHCR, and admits its share of *resettled refugees*. In contrast to convention refugees, resettled refugees are individuals who often come directly from a refugee camp and who have not entered the country individually. The size of the quota is decided annually by the Swedish government in agreement with the UNHCR. Moreover, over time, the Swedish Aliens Act of 1954 has been interpreted in a wider sense than the original Geneva Convention, creating an established practice that has enabled other refugees, beyond convention and quota refugees, to obtain permanent residence in Sweden.

Labor-market policies toward refugees have been used in Sweden since the 1970s. According to the 2015 MIPEX index (http://www.mipex.eu/), Sweden scored the highest out of all European countries and Canada on all six indicators studied, including the labor-market access indicator for immigrants and ethnic minorities. The main elements in the labor-market integration programs over recent decades have remained the same—language training, civic orientation, and labor-market activities—and are provided by either the municipalities or the labor-market authorities (since 2010). The duration of the program has been about 2 years and is financed by the government.

Program, in studied period, include the fact that housing is negotiated by the regional authorities, mainly in smaller municipalities with an abundance of housing, so that individuals can begin their introductory program. Resettled refugees are housed upon arrival by the Migration Board, which has negotiated special arrangements with a number of municipalities for both housing and integration training. However, given the shortage of housing in the larger municipalities, these refugees often end up in smaller ones (Bevelander and Pendakur, 2009). Of note is the fact that, under Swedish immigration regulations, the relatives of refugees have the right to reunion migration, too. The Swedish government, through the Swedish Red Cross, also finances the travel costs associated with reuniting relatives.

## Earlier Studies

The increase in the number of people seeking asylum has had a profound effect on European countries, not the least on Sweden, where approximately half of the settling immigrants over the last 30–40 years were refugees or their families. Whereas, a large body of literature is available on the economic integration of immigrants in host countries, far fewer studies have been conducted on the economic integration of refugees.

A number of studies in the US, Canada, the UK, the Netherlands, Denmark, Norway, and Sweden have specifically focused on the labor-market integration of refugees. The picture that this research paints is that, compared to other immigrant groups, refugees generally have lower employment rates, particularly soon after their arrival in the host country. However, over time, refugees “catch up” and show similar employment levels as other non-economic immigrant categories (de Vroome and van Tubergen, 2010; Bevelander, 2011; Hatton, 2011), although they have lower levels compared to labor migrants (Yu et al., 2007).

Theoretically, it is assumed that refugees, like other non-economic immigrants, are less favorably selected compared to labor (economic) immigrants (Borjas, 1987; Chiswick, 2008; Dustmann et al., 2017). Refugees arrive under different, and often difficult, circumstances, have not migrated primarily for labor-market reasons and are admitted according to other (non-economic) criteria, which appears to affect their labor-market integration. Both the migration and the admissions processes can be lengthy and cumbersome. Health issues and the loss of human capital can hinder individuals' adaption to the labor market of a new country. Moreover, once accepted, whether refugees and family-reunion migrants obtain permanent or temporary residence can also affect their investment in the host language and receiving-country-specific human capital and their labor-market integration process (Hainmueller et al., 2016; Dustmann et al., 2017).

Studies that focus on the employment trajectories of government-assisted refugees, asylum-seekers, and family-reunion immigrants in Sweden conclude that the differences inferred can be the product of integration policies that vary by entry category. They also point to possible differences in access to social capital and in mobility choice. Government-assisted refugees are often located in municipalities in which housing is available but where employment opportunities are scarce. Asylum-seekers often have personal resources and can settle where the job prospects look the most promising. Family-reunion immigrants are likely to draw on the social capital acquired by family and friends who have already settled in the country (Bevelander and Pendakur, 2009).

For Sweden, Rashid (2009) assessed the impact of mobility on economic outcomes for refugees. He shows that internal migration generates a positive outcome in terms of higher employment levels and family income for newly arrived refugee families; this is in line with earlier research on the attractiveness of the larger and more diversified labor markets in more densely populated areas and larger cities. This is partly because refugees often move from an area with few jobs to one with greater employment opportunities (Edin et al., 2003; Damm, 2009). The internal migration of immigrants in general, and refugees in particular, is thus an important factor when it comes to their obtaining employment.

In addition to national-level datasets, a number of special surveys have been carried out that support the relationship between immigrant entry category and economic outcomes. In the case of the Netherlands, de Vroome and van Tubergen (2010) found that host-country-specific education, work experience, language proficiency and contacts with natives were positively related to the likelihood of obtaining employment and occupational status. In another study on the Netherlands, Bakker et al. (2013) showed that post-migration stress or trauma affects refugees' labor-market integration. Survey data from a sample of 400 refugees in the United Kingdom point to the fact that policies which restrict access to the labor market also have a negative impact on refugees' employment probabilities (Bloch, 2007).

Using the Longitudinal Survey of Immigrants to Canada to compare the labor-force participation and earnings of differing categories of immigrants 2 years after their arrival, Aydemir (2011) concluded that refugees have lower participation rates than family-reunion immigrants but that their earnings are about the same. Assessment of economic outcomes in the United States has shown that refugees have lower earnings than other categories of intake but that this difference can, at least partially, be explained by differences in language ability, schooling, level of family support, mental health, and residential area. However, a gap remains even after controlling for these factors (Connor, 2010). Studies for Norway and Denmark show that refugees and family members have an initial promising increase in employment integration but a subsequent leveling out and even a reverse process after about 10 years (Bratsberg et al., 2017; Schultz-Nielsen, 2017). These studies underscore the heterogeneity within admission class, country of origin and schooling as explanatory factors for labor-market success.

Many of the studies referred to above on the differences between refugees and economic migrants have concluded that refugees are in a disadvantaged position. However, there are also discrepancies in the results of these studies: some show that refugees perform as well as other non-economic immigrants, and some that the differences are small, while others argue that the gap is substantial. However, these studies are all based on comparisons between groups *in one country*, not between countries. In Bevelander and Pendakur (2014) this problem is overcome by studying the economic integration of non-economic migrants. Directly comparing two countries and the same refugee groups, as well as admission class, provides additional insights. In their study, asylum-seekers who subsequently obtain refugee status, resettled refugees and family-reunion migrants, all of whom are non-economic immigrants, are compared in both countries. The results show that, after controlling for other variables, the probability of being employed is roughly the same in Canada and in Sweden, whereas the difference in earnings between the countries is greater and favors Canada. Additional insights from this study are that differences between intake categories are smaller in Sweden than in Canada. The authors argue that this could be due to the provision of services and programs to all categories in Sweden yet only to resettled refugees in Canada. Thus, while the employment rates are comparable between the two countries, Canada may offer greater opportunities for upward earnings mobility than Sweden. Maybe the larger wage dispersion in Canada relative to Sweden could be a possible explanation for this result.

Summarizing and in line with Chin and Cortes (2015) the research on refugee labor-market integration clearly indicates that refugees are at a disadvantage upon arrival in the host country due to unfavorable selection, loss of skills, and the lesser transferability of earlier skills compared to other migrants (see also Luik et al., 2018 for Sweden). Besides, lengthy asylum procedures negatively affect the possibilities for investing in host-country human capital. Investment in human capital by refugees as well as through labor-market policies directed at refugees, including language training, could initially overcome their difficulties in entering the labor market and lead to an adaptation in economic terms relative to other migrants and natives. However, refugees' relatively worse health due to their earlier experiences could mean that, overall, they never do “catch up” with other migrants and natives in the labor market.

In line with the above, we propose the following. Our first expectation is that there will be a heterogeneous pattern of employment integration by gender and country of birth. Our second expectation is that, after controlling for demographic and human-capital characteristics, both refugee *male* and *female* employment probability will be low in the first year after arrival although this will subsequently increase—indicating a “catch up”—or decrease, representing a “falling behind.”

## Data and Descriptive Statistics

### Data

Our analysis uses administrative data from the STATIV database of Statistics Sweden for the years 1998–2012. This database contains yearly basic demographic and socio-economic information on every legal resident in Sweden. Focusing on the labor-market outcome “employment status” for refugees^3^, we observe individuals between the age of 25 and 64 from Year 1 to Year 12–14 since arrival. While employment status is a well-established labor-market outcome, it is noteworthy that its scope is limited to the extensive margin of labor-market participation. Hence, it does not capture whether the employment is self-employed, part-time, blue- or white-collar, high- or low-paid or particularly stable. What we can show, however, is that the employment rate remains very similar if we condition it on refugees having earned at least the national minimum income. Our group of refugees includes individuals who are uniquely identifiable as either being a quota refugee or an individual seeking protection and receiving legal permanent residency^4^. In order to distinguish this group of refugees from other types of immigrant with respect to mean characteristics, employment, and outmigration, we initially also include EU28, non-EU28 labor, non-EU28 student and non-EU28 family migrants. Ultimately, this results in, respectively, 151,089 and 107,136 pooled observations on refugees and the remaining migrant groups with employment information. In the main analysis regarding the employment path of refugees, we then limit the sample to refugees who remained in Sweden throughout the sample period (on average roughly 87%; see Table 1).

**Table 1:** Mean characteristics by admission status.

	**EU 28**	**Non-EU 28**			
		**Student**	**Labor**	**Family reunification**	**Humanitarian**
**Employment status**					
Employed	0.52	0.07	0.53	0.38	0.46
Employed (>50 k)	0.51	0.07	0.52	0.37	0.46
**Socio-demographics**					
Male	0.62	0.71	0.78	0.33	0.63
Couple	0.45	0.35	0.62	0.68	0.68
Single	0.45	0.64	0.34	0.14	0.18
Children	0.82	0.18	0.66	1.62	1.64
**Human capital**					
Some college	0.54	0.42	0.55	0.38	0.32
**Municipality**					
Stockholm	0.42	0.40	0.52	0.38	0.28
Gothenburg	0.18	0.15	0.12	0.18	0.19
Malmö	0.15	0.26	0.10	0.14	0.15
**Migration-related**					
Swedish citizenship	0.00	0.01	0.01	0.01	0.03
Age at migration	31.70	29.43	34.41	32.19	32.79
Year of arrival	1999.04	1999.08	1999.09	1999.02	1999.03
Staying 12+ years	0.51	0.04	0.43	0.66	0.87
*N*	35,096	1,620	3,131	67,289	151,089
% of sample	13.59	0.01	0.01	26.06	58.51

In line with related studies from Denmark (Schultz-Nielsen, 2017) and Norway (Bratsberg et al., 2017), our strategy is to exploit the rich Swedish register data, including information on admission status upon arrival and country of origin. In this way, we avoid using less-assured measures based on a combination of country of origin and year of arrival or even self-reported reasons for migration. As we pool all those who arrived in either 1998, 1999, or 2000 as refugees and observe them from Year 1 up to a maximum of 12–14 in the years 1998–2012, we avoid posing strong assumptions with respect to cohort differences and age. While the cohort approach facilitates a longitudinal integration analysis, it does not reveal whether the evidence is representative for the different cohorts. For instance, there might be marked differences compared to the cohorts who arrived after the immigration reform in 2016 which limited permanence of stay and potential family reunion.

In our descriptive analysis and later regression adjustment, we make use of a rich set of controls such as age, sex, level of schooling (seven levels from less than lower-secondary schooling to postgraduate degree level), marital status (couple, singe, divorced, widowed), number of children, municipality of residence (Stockholm, Gothenburg, Malmö, Other), country of birth and years in Sweden. The control “Years in Sweden” starts in the year in which individuals obtained their residence permit^5^. Of particular interest, the dataset also contains information on the immigrant entry category, which makes it possible to track the employment integration pattern of refugee groups over time.

### Descriptive Statistics

Before we conduct the aforementioned detailed analysis of different major refugee groups, it is instructive to describe the overall group and highlight how it differs compared other groups of immigrants who were admitted to Sweden as EU citizens, non-EU students, labor or family migrants. Table 1 shows the pooled means from the years 2001 to 2012. It immediately becomes clear that there is marked heterogeneity with respect not only to employment but also to the potential determinants of employment such as age, sex, marital status, children, education^6^, residence, age at migration, and percentage of those who have been in Sweden for at least 12 years. For instance, labor migrants are relatively likely to be employed and predominantly male and to have a low average number of children; 50% of our observations are recorded in Stockholm. Compared to this, refugees are less likely to be employed, less likely to be male, more likely to be married, have on average more children and are more spatially dispersed throughout Sweden.

**Figure 1:**
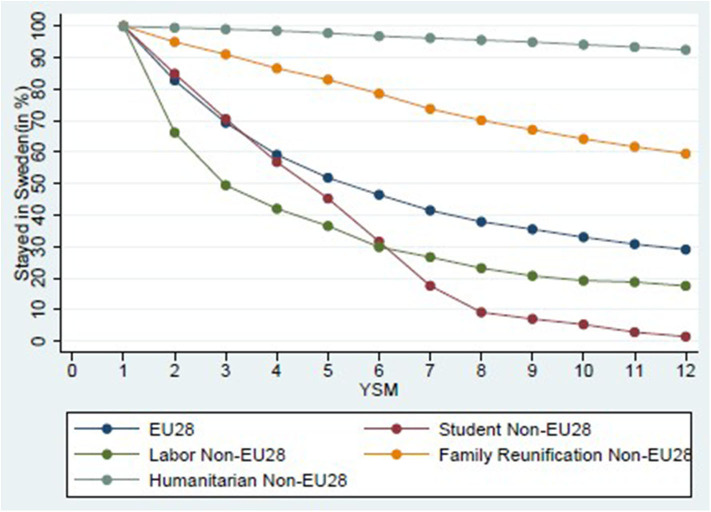
Outmigration by category.

As indicated by the last row in Table 1, admission classes seem to differ considerably with respect to outmigration. This is confirmed by Figure 1, which plots the remaining stock of the 1998–2000 immigration cohort over the years since migration. Outmigration is a threat to any cohort integration study, as a narrowing gap might be driven by negatively selected outmigration. If, instead, the focus is on the population remaining in the country, this cannot be considered as representative of the original cohort. It would therefore be good to have as little outmigration as possible. By focusing on the refugee cohort, however, we are studying a group with comparably little outmigration. Our data suggest that, for this Swedish immigrant cohort, after 3 years, only 50 and 70% of the non-EU28 labor and EU28 immigrants remain in Sweden. In contrast to this, almost the entire entry cohort of humanitarian immigrants still resides in Sweden. This pattern is reinforced over time so that, after 12 years, roughly 90% of the original humanitarian but only20 % of labor immigrants have stayed in Sweden.

The presented pattern is in line with related empirical evidence for the UK (Dustmann and Weiss, 2007) and Norway (Bratsberg et al., 2017) and highlights the need to understand outmigration patterns and immigrant heterogeneity, as well as the economic and fiscal importance of the refugee group (Bratsberg et al., 2017).

From now on and throughout the main part of our study, we limit our analysis to refugees. In particular, we focus on the eight main source countries of Iraq, Iran, Afghanistan, and Syria (the Middle East), Somalia, Ethiopia, and Eritrea (East Africa) and Bosnia-Hercegovina (Europe). While the literature (Dustmann et al., 2017) is acknowledging heterogeneity between admission statuses, the evidence with respect to heterogeneity between refugees is limited. This is surprising as there is no reason to assume homogeneity for individuals who seek protection for a variety of reasons and within very diverse contexts.

Like Tables 1, 2 reports the mean statistics for refugees by country of origin. Again, a striking heterogeneity with respect to employment, socio-demographic characteristics and outmigration becomes apparent. The mean employment share is very similar for Iraqi, Iranian, Afghan, and Syrian refugees at around 40–44 %. In contrast to this, only 26% of Somali and over 60% of Ethiopian, Eritrean, and Bosnian refugees are employed. The pattern holds for employment with a minimum income of 50,000 Swedish kronor (about 6,000 US dollars). Despite their similarity in employment, Iranians and Iraqis differ markedly with respect to education, sex, marital status, and residence. The same can be observed for Eritreans and Ethiopians.

**Table 2:** Mean characteristics.

	**All**	**Iraq**	**Iran**	**Afghanistan**	**Somalia**	**Syria**	**Ethiopia**	**Eritrea**	**Bosnia**
**Employment status**									
Employed	0.44	0.40	0.42	0.41	0.26	0.44	0.65	0.64	0.61
Employed (>50 K)	0.43	0.39	0.42	0.40	0.25	0.44	0.65	0.64	0.61
**Socio-demographics**									
Age	39.26	39.25	39.84	39.58	35.70	41.06	38.43	40.38	39.39
Male	0.65	0.73	0.54	0.72	0.43	0.52	0.62	0.58	0.49
Couple	0.72	0.75	0.66	0.82	0.58	0.68	0.45	0.58	0.68
Single	0.16	0.14	0.17	0.10	0.25	0.17	0.37	0.27	0.20
Children	1.64	1.66	1.71	2.23	1.80	1.71	0.77	1.12	1.46
**Human capital**									
Some college	0.33	0.39	0.25	0.40	0.09	0.31	0.29	0.16	0.17
**Municipality**									
Stockholm	0.32	0.37	0.25	0.43	0.41	0.41	0.58	0.52	0.08
Gothenburg	0.20	0.18	0.21	0.19	0.27	0.13	0.19	0.21	0.23
Malmö	0.14	0.13	0.05	0.18	0.05	0.06	0.05	0.01	0.23
**Migration-related**									
Citizenship	0.57	0.59	0.57	0.40	0.31	0.64	0.58	0.55	0.60
Age at arrival	32.67	32.67	33.28	32.93	29.11	34.38	32.00	33.85	32.77
Year of arrival	1999.03	1999.13	1998.79	1999.49	1998.75	1999.06	1998.93	1998.88	1998.70
Stay ≥ 12 years	0.86	0.86	0.85	0.83	0.68	0.87	0.84	0.92	0.90
*N*	104,791	65,141	8,424	5,359	3,216	1,886	1,539	1,017	18,209
In % of sample		62.16	8.04	5.11	3.07	1.80	1.47	0.97	17.38

**Figure 2:**
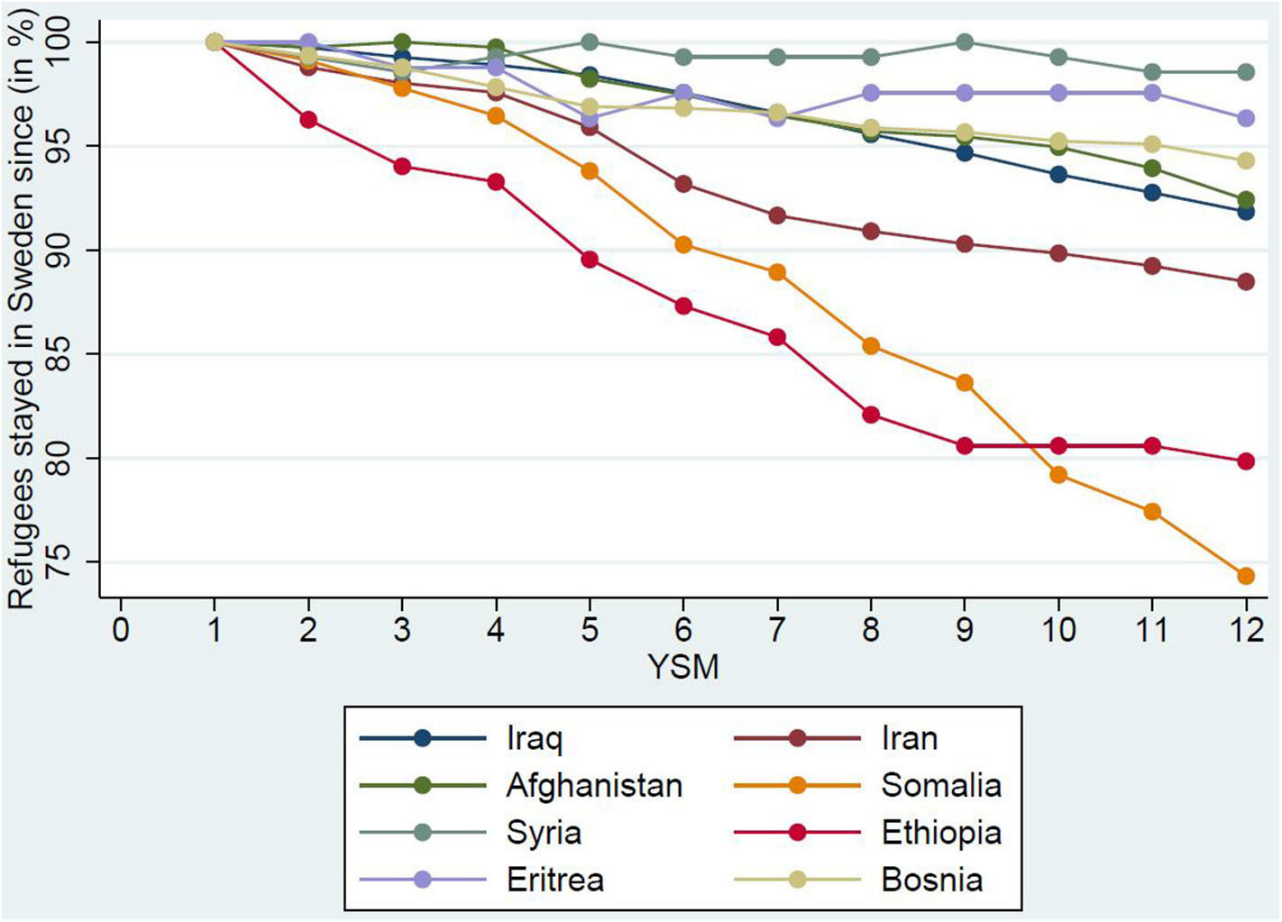
Outmigration by country of birth.

Turning to outmigration, we again visualize the remaining share of the entry cohort. Figure 2 suggests that, after 5 years in the country, some refugee groups—most notably Somalis and Ethiopians—return or onward migrate. This is in line with research highlighting the onward migration of (naturalized) Somalis from Sweden to the United Kingdom due to the right of free movement, a critical mass of Somalis in the UK and a self-proclaimed “nomad” culture (Osman, 2012). In the case of immigrants from Somalia, this outmigration results in lower employment rates, as there is evidence of a positive selection into outmigration with respect to self-employment (Carlson and Galvao Andersson, 2017).

Consequently, focusing on humanitarian immigrants (and even the geographical region such as East Africa) alone will not be sufficient to avoid an attrition bias in our study of longitudinal integration^7^. Hence, in our main analysis of employment integration, we focus on selected individuals who stay throughout our data window.

## Employment Integration

**Figure 3:**
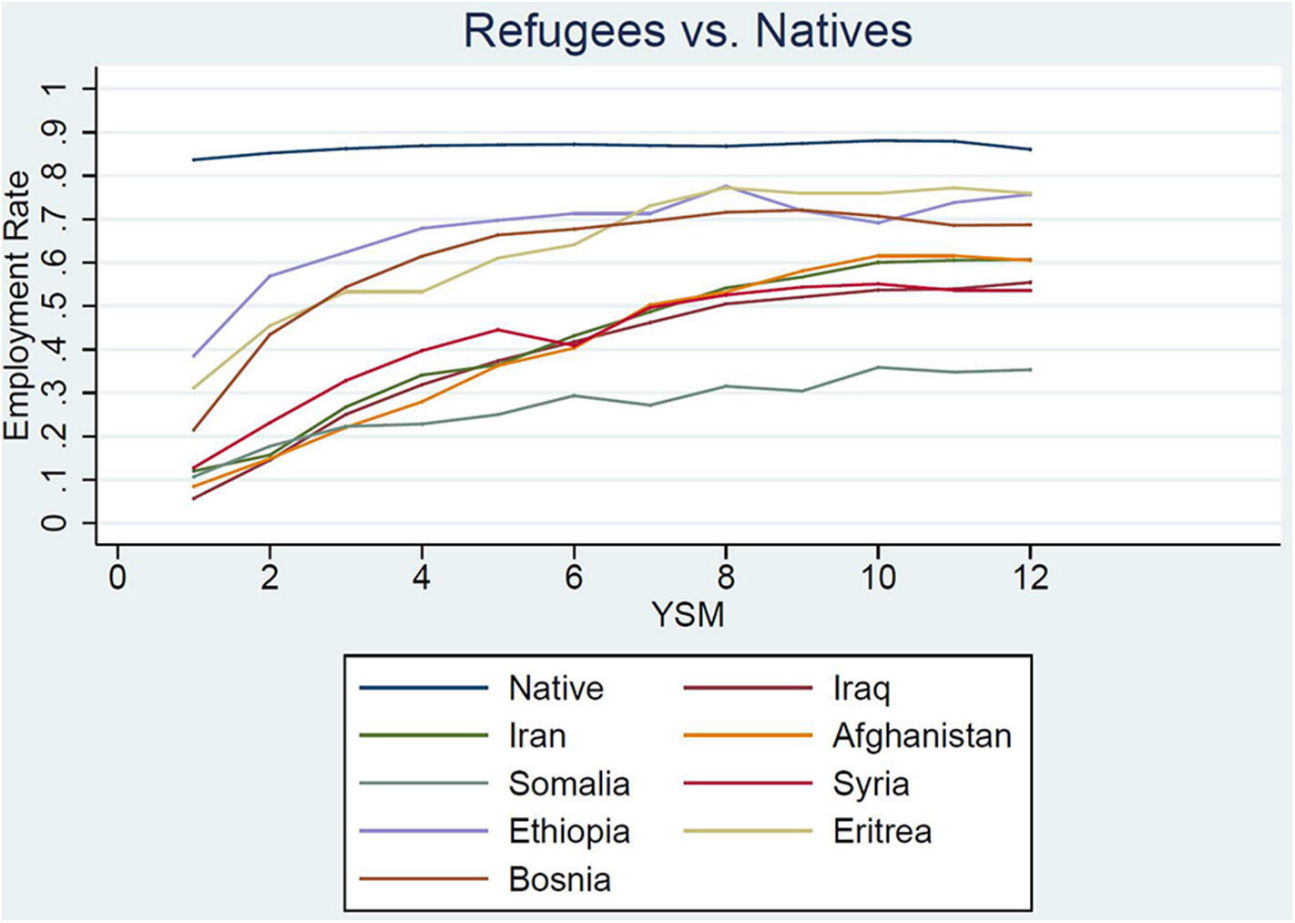
Employment rate by country of birth.

In Figure 3 we visualize the average employment for each refugee group from between 1 and 12 years since migration, conditional on observing the individual over the entire time span. In addition, for comparability, we also plot average employment figures for a native control group, based on the same age filter in the year 1999 and followed over the same window of time. While the native share of those employed is very stable at around 88%, each refugee group follows a more or less steep increase after a low entry average, and hence slowly although not fully catches up, which is in line with the model of human-capital investment and integration in Duleep and Regets (1999).

Focusing on the initial employment likelihood after 1 year in the country, we can identify a group of very low and medium–low employment integration. Iraqis, Iranians, Afghans, Syria, and Somalians only had an employment share of around 10 %, whereas already 20, 30, and 40% of Bosnians, Eritreans, and Ethiopians, respectively, have been employed after 1 year in the country. In contrast to the prediction by Duleep and Regets (1999), however, the groups with the lowest relative employment upon arrival—and hence the highest incentive to invest to catch up—do not experience a steeper employment growth. Again Bosnians, Eritreans, and Ethiopians increase their employment share the most up to the seventh and eighth years since migration. After that the employment share seems to stagnate at between 70 and 80%. Migrants from Middle Eastern countries are on a slower growth path up until 10–12 years since migration, reaching a 50–60% employment rate, whereas Somalian refugees also improve their relative employment but at a substantially slower rate.

**Figure 4:**
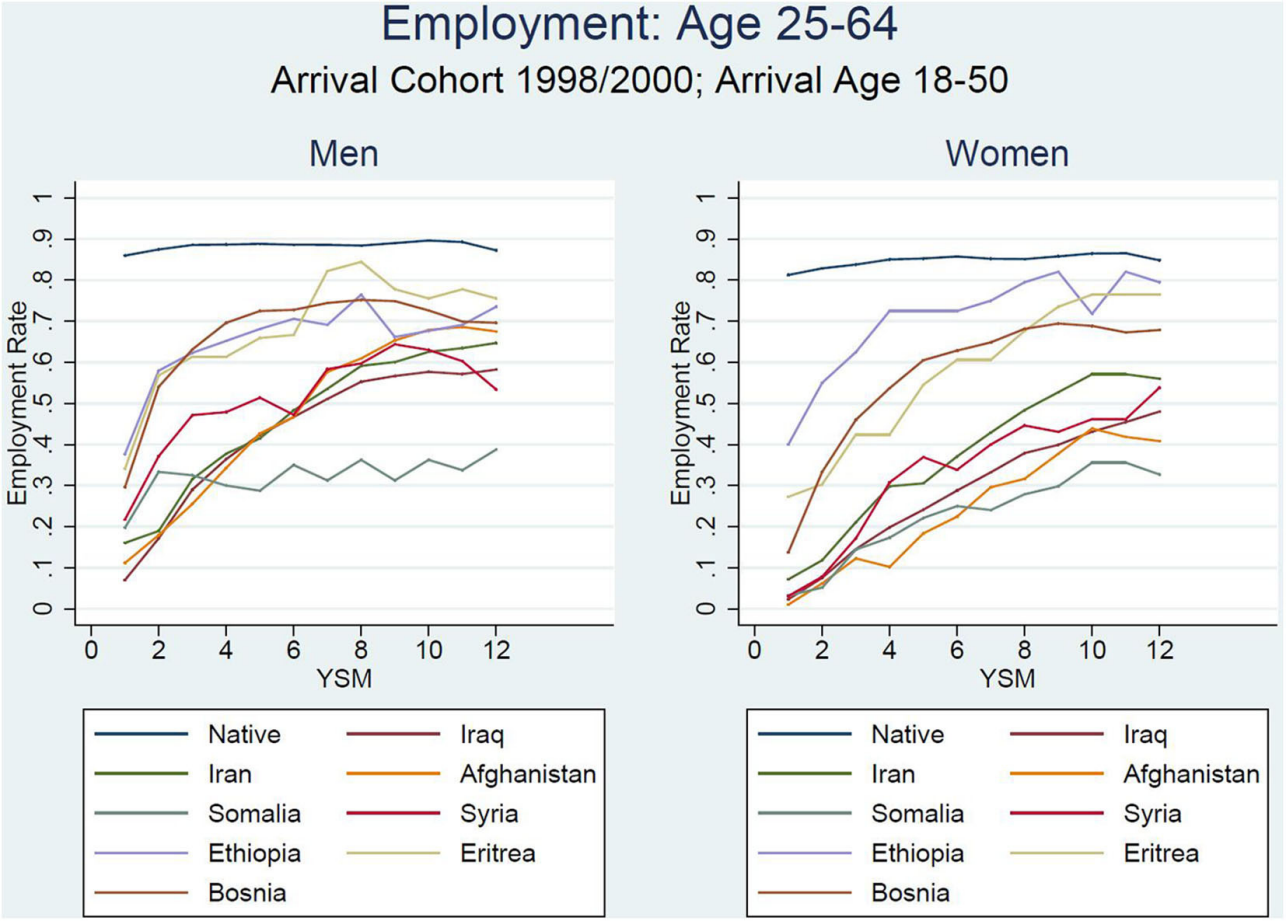
Employment rate by country of birth and gender.

Splitting the sample into male and female refugees causes a few interesting patterns to emerge (Figure 4). First, the two groups seem to prevail in both subsamples while being more marked among women. Second, for all origin groups except the Ethiopians, the initial female employment shares were lower than those of their male counterparts. Both groups are catching up to remarkable yet varying degrees. For men from Bosnia, Ethiopia, and Eritrea, the employment growth plateaus after roughly 6 years since migration. Among these men, only the employment share of Bosnian men who entered Sweden as refugees decreases from the seventh year since migration. The same hump shape can be observed for Syrians on a much lower employment level. For male refugees from Iraq, Iran, and Afghanistan, growth is slower but continues until the 12th year since migration; for their counterparts from Somalia, the growth almost stagnates. While the employment path of Bosnian refugees is comparable to evidence for refugees in Norway (Bratsberg et al., 2017), we do not observe this for the majority of refugees to Sweden. It is noteworthy that the drop is absent for most of the groups despite the confounding Great Recession.

As in the Norwegian case, however, we find a slower but continuous catch-up for female refugees within the first 12 years. However, our data even suggest that the employment share in Year 12 is slightly higher for Ethiopian and Eritrean women compared to their also comparatively assimilated male counterparts.

## What Drives the Gaps by Country of Origin?

In order to design economic policy suitable to this integration heterogeneity, naturally we would like to identify the underlying drivers of the gaps between refugees and between refugees and natives. As a first step into this direction, we want to ascertain whether or not differences in observables related to socio-demographics and latent human capital are a critical factor. Regarding labor-market outcomes, human capital in particular is an intuitive first candidate to test, as it is the key success factor in traditional human-capital theory. Following this argument, one would expect differences in the employment paths to decrease as soon as these groups are rendered comparable with respect to human capital. Note that, interestingly, our descriptive table is at odds with this prediction, as comparably low shares of college attendance coincide with high employment shares. A remaining gap suggests unobserved heterogeneity related to country of origin. It is noteworthy that this can still be related to human capital if it differs by origin or transferability, or even discrimination, social networks, trauma or source-country welfare incentives. In this paper, however, we focus on the role of the above-mentioned observable differences and leave more detailed explanations for future research.

In particular, we estimate a male and a female linear probability model of employment for natives and refugees alike. In order to derive an assimilation path relative to native employment, we include a country-of-origin indicator and interact it with a third-order polynomial of years since migration in the fashion of works such as that by Bratsberg et al. (2014). At the same time, we fully interact the group indicator with a third-order polynomial of age and include an error term and a constant. As our variable of interest is time-invariant, we do not include any individual fixed effects. In order to make the groups comparable, however, we also control for human capital through educational attainment, marital status, number of children, contextual municipality (Stockholm, Gothenburg, Malmö, Other and year fixed effects. The latter two capture the effects of local labor-market disparities and macro shocks. Note that a prior decision to move to a regional labor market is an endogenous choice which could also vary by refugee group. In order to capture regional macro developments, we also fully interact municipality and year^8^. It is noteworthy that we therefore assume equal year fixed effects and association with the business cycle for all groups (Bratsberg et al., 2014). The detailed regression output for men and women can be seen in Supplementary Tables 4, 5.

While this needs to be kept in mind, it should be a less-severe issue through the same admission process. Moreover, we can rule out major differences with respect to the institutional and legal framework (processes) and the related uncertainty which has been shown to impede integration (Dustmann et al., 2017).

**Figure 5:**
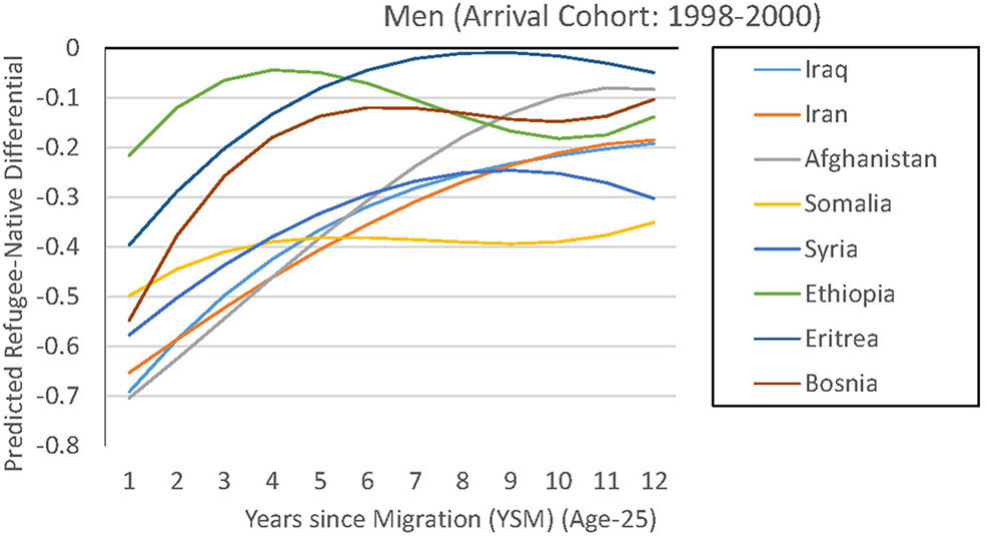
Regression-based prediction of refugee employment differential (men).

As we are mainly concerned with the remaining heterogeneity, we again report the resulting employment paths in two figures. Technically, each line is a model prediction for a specific immigrant group, where we keep all values constant at the mean except years since migration and age. For instance, in Figure 5 we can see that Eritrean male refugees assimilated in terms of employment from a gap of −40 to −5% points (between 1 and 9 years since migration). The regression-adjusted employment path is hence on a higher level than its unadjusted counterpart. The remaining gap is not statistically different from zero, as can be seen in Supplementary Table 3, which reports the predicted immigrant–native employment differentials for four, 8 and 12 years since migration, together with the underlying standard errors^9^. The same can be observed for the group from Bosnia-Hercegovina. These are examples for groups that experience strong employment growth despite less-favorable observable characteristics, so that they are unlikely to be the main gap driver. An interesting case in the male sample is Ethiopian refugees, whose observable differences to natives seem to be the main explanation for their initially low employment level. Adjusting these differences, they are the group with the highest employment share in the first year since migration. Overall, for them and for the remaining groups, the regression adjustment decreases the gap with natives and other refugees over the entire timeframe but can only be considered a smaller part of the explanation, as gaps, ranking, and growth profiles remain for most of the groups.

**Figure 6:**
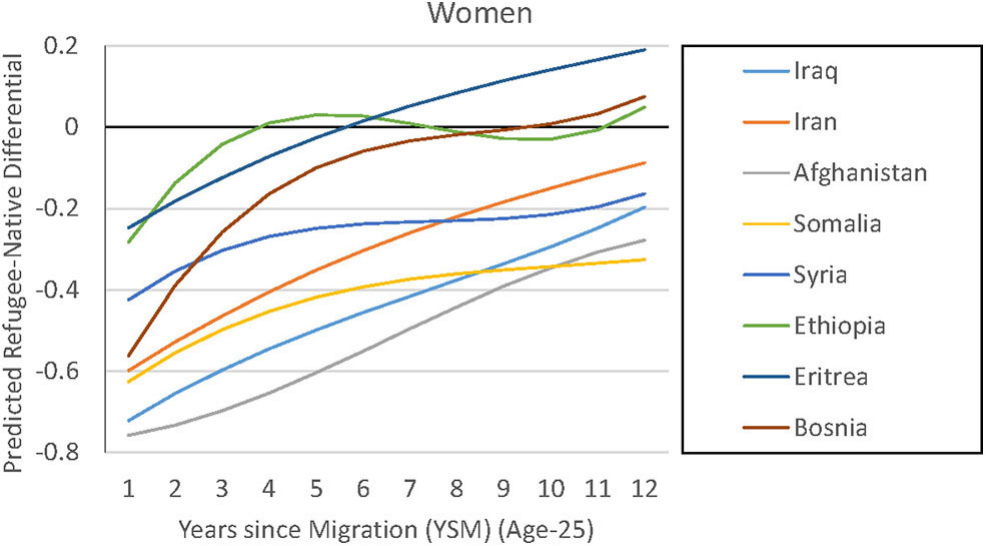
Regression-based prediction of refugee employment differential (women).

In our female sample, we can present similar observations (Figure 6). In fact, making observables comparable means that Ethiopian, Eritrean, and Bosnian women are climbing to a higher employment path—this results in an employment share that is even higher than that of their Swedish counterparts. Notably smaller gaps with natives can also be detected for female refugees from Syria and Somalia. Two more observations can be made: first, there is still a group of refugees who start at a very low employment level. Afghan and Iraqi women start at an employment share that is up to 80% points lower than that of natives. Moreover, the employment path is less hump-shaped and more linear for a lot of groups, which is not in line with decreasing incentives to invest in host-country-specific human capital. Again, we can see that these observable characteristics do play a role but that they cannot account for group differences among refugees or for the entire gap between them and the natives. Notable exceptions are the group of Ethiopian, Eritrean, and Bosnian women.

## Concluding Discussion

Globally growing numbers of asylum-seekers have also found their way to Sweden and put refugee labor-market integration high on the political agenda. Just as in many other European countries, the employment levels of immigrants in general are lower than for natives (OECD, 2017). Immigration to Sweden over recent decades has, to a considerable extent, been by refugees and could partially explain the native–immigrant employment gap. However, few studies have exclusively focused on possible dissimilarity in employment integration by country of origin and gender in explaining refugees' overall lower employment levels. In order to shed some light on this highly important issue, we have (a) studied the overall employment integration heterogeneity by country of origin, (b) described whether refugee groups are able to close the gap or fall behind relative to Swedish natives' employment levels and (c) provided insights into whether refugee differences with respect to demographics and human-capital characteristics could be a potential driver of heterogeneity.

In line with earlier studies—Bratsberg et al. (2017) for Norway, Schultz-Nielsen for Denmark (2017) and Bevelander (2011) for Sweden—our descriptive cohort analysis has shown that initial employment levels for both males and females and a number of groups of refugees are low. Female and male individuals from Iran, Iraq, Somalia, Syria, and Afghanistan do reach employment levels of roughly 10–20% on arriving in the country, whereas those from Bosnia, Ethiopia, and Eritrea have ~30–40% in employment levels on entering the labor market. Subsequently, these two groups show different employment integration patterns. Over time, the “catch up” process is somewhat faster and more extensive for the Bosnian, Eritrean, and Ethiopian group than for the Asia/Somalia group. However, no “falling behind” is evidenced by our analysis. All groups do increase their employment levels but from different starting points and at varying speeds.

Our further analysis, controlling for observable demographic and human-capital characteristics, shows that all refugee groups—both males and females—gradually increase their employment probability over time. The underlying estimated model has, among other effects, the positive effect of education, being in a couple, having children and being in employment in Stockholm. While differences between groups decrease after regression adjustment, the pattern of heterogeneity remains intact and is non-negligible. Notably, however, both male and female refugees from Bosnia and Eritrea, as well as Ethiopian women, have close to, the same or an even higher probability of being employed as do their Swedish counterparts. These results indicate that, for these latter groups, observable human-capital and context characteristics explain the—comparably smaller—difference in employment levels, although the time to parity takes about 4–8 years of living in the country. Considering that natives tend to be less likely to be in a couple, have less children and more dispersed across Sweden, the difference in the Swedish education distribution could be a gap driver. While, we have not conducted a detailed decomposition here, Luik et al. (2018) show that the native share of lowest education tends to be lower than in the group of refugees, whereas the share of higher education is comparable. It suggests that an on average higher education could close and even reverse the gap. Any remaining employment probability differentials for female and male refugees from Iran, Iraq, Afghanistan, Syria, and Somalia, relative to Swedish females and males, are between 10 and 30%—even after 12 years in the country.

The results found for Bosnian, Iranian, Iraqi, and Afghan refugees are on a par with earlier cross-sectional studies for this group (Bevelander, 2011; Bevelander and Pendakur, 2014). Nevertheless, the inclusion of other male and female refugee groups does show that Bosnian, Eritrean, and Ethiopian refugees of *both* sexes find their way in the Swedish labor market and reach parity with their Swedish-born counterparts. This result stands in clear contrast to those of other European studies for Norway and the Netherlands (Bakkaer, 2015; Bratsberg et al., 2017), where no refugee group reaches parity with their native counterparts. In relation to the refugee groups with lower entrance and speed rates, their employment levels are also clearly higher compared to, for example, studies from Denmark and the Netherlands (Bakkaer, 2015; Schultz-Nielsen, 2017).

Any remaining differences between refugee groups and natives as well as between refugee groups are difficult to assess within this analysis. Possible differences could be due to the fact that larger proportions of the refugee groups studied have gained access to Sweden under the UN resettlement program. Earlier studies have shown that resettled refugees have a slower employment integration rate compared to refugees who seek and are granted asylum at the border (Bevelander, 2011). The argument is that those who have the ability and resources to travel all the way to Sweden and seek asylum are positive selected compared to those who are chosen from refugee camps around the world, and that resettled refugees will probably have fewer networks to help them in the new country (Hatton, 2011). These two arguments also apply to the overall group of refugees, as emigration-inducing shock can affect either the entire population or a selected subgroup (Chin and Cortes, 2015). The latter can differ with respect to labor-market skills and the extent of their local social networks. As in the case of Somali immigrants, this might not only accelerate integration but also lower onward migration (Osman, 2012). Other group variations could lie in the differential transferability of human capital through, for instance, differences in the origin-country local educational system, job, and skill distribution, language or even historical ties (i.e., through developmental work). This, again, might affect the duration of and uncertainty during the asylum process, as well as the timely investment in Swedish human capital (Dustmann et al., 2017). Naturally, and finally, the emigration-inducing shock differs for each group, so that human and health capital might have been diminished to different degrees.

## Author Contributions

All authors listed have made a substantial, direct and intellectual contribution to the work, and approved it for publication.

## Conflict of Interest

The authors declare that the research was conducted in the absence of any commercial or financial relationships that could be construed as a potential conflict of interest.

## Footnotes

^1^The native–immigrant employment gap is about 14% in Sweden and only 3% in the EU(28).

^2^However, it is important to note that our population of immigrants might be slightly over-represented due to the lack of deregistration upon emigration.

^3^We use the standard European definition of employment—being employed at a minimum of one base value during the month of September. The base employment value is defined as an annual income of at least 44,000 Kronor (equivalent to the social security payment). It is generally used to calculate the value of unemployment insurance.

^4^During this period, all asylum-seekers, when being accepted as refugees, received permanent residence permits. Family members arriving later are not categorized as refugees and are not included in the analysis.

^5^Earlier studies have shown that limited time in the asylum procedure increases the labor-market integration process (Bevelander and Pendakur, 2009).

^6^Education is measured either when individuals are assessed by the employment service during the introductory program or after re-education in Sweden. In the introductory program, foreign qualifications and certificates are evaluated and converted to Swedish standards. This probably increases the transferability of foreign qualifications.

^7^A breakdown by sex can be found in Supplementary Tables 1, 2. The tables highlight marked gender differences within and across countries. As a consequence, we conduct our main analysis for men and women separately.

^8^Note that we are not interested in identifying the precise effects of these determinants, which is why we refrain from discussing endogeneity related to factors such as residence.

^9^The detailed coefficients of the male and female estimations are available on request.

## References

[B1] Aydemir A. (2011). Immigrant selection and short-term labor market outcomes by visa category. J. Popul. Econ. 24, 451–475. 10.1007/s00148-009-0285-0

[B2] Bakkaer L. (2015). Seeking sanctuary in the Netherlands, opportunities and obstacles to refugee integration (Ph.D. thesis). Erasmus University, Rotterdam, the Netherlands.

[B3] Bakker L. Dagevos J. Engbersen G. (2013). The importance of resources and security in the socio-economic integration of refugees: a study on the impact of length of stay in asylum accommodation and residence status on socio-economic integration for the four largest refugee groups in the Netherlands. J. Int. Migr. Integr. 15, 431–448. 10.1007/s12134-013-0296-2

[B4] Bevelander P. (2011). The employment integration of resettled refugees, asylum claimants, and family reunion migrants in Sweden. Refugee Survey Q. 30, 22–43. 10.1093/rsq/hdq041

[B5] Bevelander P. Pendakur R. (2009). “The employment attachment of resettled refugees, refugees and family reunion migrants in Sweden,” in: *Resettled and Included? The Employment Integration of Resettled Refugees in Sweden*, eds P. Bevelander, M. Hagström, and S. Rönnqvist (Malmö: Malmö University, 227–245.

[B6] Bevelander P. Pendakur R. (2014). The labor market integration of refugee and family reunion immigrants: a comparison of outcomes in Canada and Sweden. J. Ethnic Migr. Stud. 40, 689–709. 10.1080/1369183X.2013.849569

[B7] Bloch A. (2007). Refugees in the UK labor market: the conflict between economic integration and policy-led labor market restriction. J. Soc. Policy 37, 21–36. 10.1017/S004727940700147X

[B8] Borjas G. J. (1987). Self-selection and the earnings of immigrants. Am. Econ. Rev. 77, 531–553. 10.3386/w2248

[B9] Bratsberg B. Raaum O. Røed K. (2014). Immigrants, labor market performance, and social insurance. Econ. J. 124, 644–683. 10.1111/ecoj.12182

[B10] Bratsberg B. Raaum O. Røed K. (2017). Immigrant labor market integration across admission classes. Nordic Econ. Policy Rev. 520, 17–54. Available online at: http://www.diva-portal.org/smash/get/diva2:1090694/FULLTEXT01.pdf

[B11] Carlson B. Galvao Andersson G. (2017). “Competition or cooperation? Somalinomics in the UK,” in MIM Working Paper Series 17/9 (Malmö: University of Malmö).

[B12] Chin A. Cortes K. E. (2015). “The refugee/asylum seeker,” in The Handbook on the Economics of International Migration, Volume 1, eds B. Chiswick, and P. Miller (Amsterdam, Elsevier), 585–858.

[B13] Chiswick B. R. (2008). “Are immigrants favorably self-selected? An economic analysis,” in Migration Theory, Talking Across Disciplines, eds C. Brettell and J. Hollifield (New York, NY: Routledge, 63–82.

[B14] Connor P. (2010). Explaining the refugee gap: economic outcomes of refugees versus other immigrants. J. Refugee Stud. 23, 377–397. 10.1093/jrs/feq025

[B15] Damm A. P. (2009). Ethnic enclaves and immigrant labor market outcomes: quasi-experimental evidence. J. Labor Econ. 2, 281–314. 10.1086/599336

[B16] de Vroome T. van Tubergen F. (2010). The employment experience of refugees in the Netherlands. Int. Migr. Rev. 44, 376–403. 10.1111/j.1747-7379.2010.00810.x 22171361

[B17] Duleep H. O. Regets M. C. (1999). Immigrants and human capital investment. Am. Econ. Rev. 89, 186–191. 10.1257/aer.89.2.186

[B18] Dustmann C. Fasani F. Frattini T. Minale L. Schönberg U. (2017). On the economics and politics of refugee migration. Econ. Policy 32, 497–550. 10.1093/epolic/eix008

[B19] Dustmann C. Weiss Y. (2007). Return migration: theory and empirical evidence from the UK. Br. J. Indus. Relat. 45, 236–256. 10.1111/j.1467-8543.2007.00613.x

[B20] Edin P. A. Fredriksson P. Åslund O. (2003). Ethnic enclaves and the economic success of immigrants: evidence from a natural experiment. Q. J. Econ. 118, 329–357. 10.1162/00335530360535225

[B21] Hainmueller J. Hangartner D. Lawrence D. (2016). When lives are put on hold, lengthy asylum processes decrease employment among refugees. Sci. Adv. 2, 1–7. 10.1126/sciadv.1600432 27493995 PMC4972466

[B22] Hatton T. J. (2011). Seeking Asylum, Trends and Policies in the OECD. London: Centre for Economic Policy Research (CEPR).

[B23] Luik M.-A. Emilsson H. Bevelander P. (2018). Explaining the male native–immigrant employment gap in Sweden: the role of human capital and migrant categories. J. Popul. Res. 35, 363–398. 10.1007/s12546-018-9206-y

[B24] OECD (2017). International Migration Outlook 2017. Paris: OECD Publishing.

[B25] Osman A. (2012). In search of green pastures. Nordic J. Migr. Res. 2, 133–140. 10.2478/v10202-011-0035-8

[B26] Rashid S. (2009). Internal migration and income of immigrant families. J. Immigr. Refugee Stud. 7, 180–200. 10.1080/15562940902935688

[B27] Schultz-Nielsen M.-L. (2017). Labor market integration of refugees in Denmark. Nordic Econ. Policy Rev. 520, 55–85. Available online at: http://www.diva-portal.org/smash/get/diva2:1090694/FULLTEXT01.pdf

[B28] Yu S. Ouellet E. Warmington A. (2007). Refugee integration in canada: a survey of empirical evidence and existing services. Refuge 24, 17–34.

