# Supplementary material for: Refugee Employment Integration Heterogeneity in Sweden: Evidence From a Cohort Analysis
Source: Front Sociol. 2020 Jul 2;5:44. doi: 10.3389/fsoc.2020.00044 (PMC8022623; doi:10.3389/fsoc.2020.00044)
Supplement: Supplementary file 1 [file Table_1.DOCX]

Table 1. Mean characteristics, men

|  | All | Iraq | Iran | Afghanistan | Somalia | Syria | Ethiopia | Eritrea | Bosnia |
| --- | --- | --- | --- | --- | --- | --- | --- | --- | --- |
| Employed | 0.47 | 0.43 | 0.46 | 0.47 | 0.32 | 0.53 | 0.61 | 0.68 | 0.66 |
| Employed(>50k) | 0.46 | 0.43 | 0.46 | 0.46 | 0.32 | 0.52 | 0.61 | 0.68 | 0.66 |
| Age | 39.26 | 39.01 | 40.66 | 40.16 | 37.33 | 40.86 | 39.31 | 39.98 | 39.53 |
| Male | 1.00 | 1.00 | 1.00 | 1.00 | 1.00 | 1.00 | 1.00 | 1.00 | 1.00 |
| Couple | 0.73 | 0.75 | 0.66 | 0.84 | 0.61 | 0.65 | 0.44 | 0.58 | 0.68 |
| Single | 0.18 | 0.16 | 0.21 | 0.10 | 0.26 | 0.24 | 0.42 | 0.33 | 0.23 |
| Children | 1.58 | 1.63 | 1.48 | 2.25 | 1.14 | 1.58 | 0.79 | 1.08 | 1.31 |
| Some college | 0.36 | 0.40 | 0.28 | 0.47 | 0.15 | 0.34 | 0.34 | 0.24 | 0.19 |
| Stockholm | 0.33 | 0.36 | 0.26 | 0.45 | 0.38 | 0.32 | 0.52 | 0.48 | 0.08 |
| Gothenburg | 0.19 | 0.18 | 0.22 | 0.19 | 0.28 | 0.15 | 0.20 | 0.24 | 0.23 |
| Malmö | 0.14 | 0.13 | 0.04 | 0.19 | 0.05 | 0.08 | 0.04 | 0.00 | 0.22 |
| Citizenship | 0.56 | 0.58 | 0.57 | 0.39 | 0.32 | 0.65 | 0.59 | 0.54 | 0.56 |
| Age at arrival | 32.69 | 32.43 | 34.14 | 33.56 | 30.88 | 34.19 | 33.02 | 33.48 | 32.92 |
| Year of arrival | 1999.04 | 1999.10 | 1998.75 | 1999.47 | 1998.83 | 1999.15 | 1998.97 | 1998.87 | 1998.69 |
| Stay ≥12 years | 0.86 | 0.86 | 0.85 | 0.85 | 0.70 | 0.88 | 0.86 | 0.91 | 0.89 |
| N | 68,514 | 47,343 | 4,548 | 3,840 | 1,368 | 974 | 954 | 585 | 8,902 |
