# Supplementary material for: Refugee Employment Integration Heterogeneity in Sweden: Evidence From a Cohort Analysis
Source: Front Sociol. 2020 Jul 2;5:44. doi: 10.3389/fsoc.2020.00044 (PMC8022623; doi:10.3389/fsoc.2020.00044)
Supplement: Supplementary file 2 [file Table_2.DOCX]

Table 2. Mean characteristics, women

|  | All | Iraq | Iran | Afghanistan | Somalia | Syria | Ethiopia | Eritrea | Bosnia |
| --- | --- | --- | --- | --- | --- | --- | --- | --- | --- |
| Employed | 0.38 | 0.29 | 0.38 | 0.25 | 0.21 | 0.35 | 0.71 | 0.59 | 0.56 |
| Employed (>50k) | 0.37 | 0.29 | 0.37 | 0.24 | 0.21 | 0.35 | 0.71 | 0.59 | 0.56 |
| Age | 39.26 | 39.88 | 38.88 | 38.10 | 34.49 | 41.28 | 36.99 | 40.93 | 39.25 |
| Male | 0.00 | 0.00 | 0.00 | 0.00 | 0.00 | 0.00 | 0.00 | 0.00 | 0.00 |
| Couple | 0.70 | 0.73 | 0.67 | 0.76 | 0.55 | 0.71 | 0.47 | 0.58 | 0.68 |
| Single | 0.14 | 0.11 | 0.13 | 0.11 | 0.24 | 0.10 | 0.30 | 0.20 | 0.16 |
| Children | 1.76 | 1.74 | 1.98 | 2.17 | 2.29 | 1.85 | 0.73 | 1.18 | 1.61 |
| Some college | 0.27 | 0.38 | 0.22 | 0.22 | 0.05 | 0.27 | 0.20 | 0.06 | 0.16 |
| Stockholm | 0.30 | 0.38 | 0.23 | 0.36 | 0.44 | 0.51 | 0.69 | 0.57 | 0.08 |
| Gothenburg | 0.20 | 0.19 | 0.20 | 0.18 | 0.25 | 0.12 | 0.17 | 0.17 | 0.23 |
| Malmö | 0.14 | 0.12 | 0.06 | 0.15 | 0.04 | 0.04 | 0.07 | 0.03 | 0.24 |
| Citizenship | 0.59 | 0.61 | 0.56 | 0.44 | 0.31 | 0.64 | 0.56 | 0.56 | 0.63 |
| Age at arrival | 32.64 | 33.28 | 32.27 | 31.33 | 27.81 | 34.58 | 30.33 | 34.34 | 32.62 |
| Year of arrival | 1999.01 | 1999.20 | 1998.84 | 1999.55 | 1998.68 | 1998.96 | 1998.86 | 1998.89 | 1998.70 |
| Stay ≥12 years | 0.86 | 0.86 | 0.85 | 0.77 | 0.67 | 0.86 | 0.81 | 0.93 | 0.90 |
| N | 36,277 | 17,798 | 3,876 | 1,519 | 1,848 | 912 | 585 | 432 | 9,307 |
