# Supplementary material for: Refugee Employment Integration Heterogeneity in Sweden: Evidence From a Cohort Analysis
Source: Front Sociol. 2020 Jul 2;5:44. doi: 10.3389/fsoc.2020.00044 (PMC8022623; doi:10.3389/fsoc.2020.00044)
Supplement: Supplementary file 3 [file Table_3.DOCX]

Table 3. Predicted immigrant–native employment gap

|  | Men | | | Women | | |
| --- | --- | --- | --- | --- | --- | --- |
| Years since migration | 4 | 8 | 12 | 4 | 8 | 12 |
| Iraq | -0.425***  (0.010) | -0.254***  (0.009) | -0.192***  (0.019) | -0.545***  (0.158) | -0.376***  (0.016) | -0.197***  (0.029) |
| Iran | -0.462***  (0.041) | -0.269***  (0.033) | -0.185***  (0.039) | -0.405***  (0.038) | -0.220***  (0.039) | -0.088*  (0.046) |
| Afghanistan | -0.462***  (0.041) | -0.178***  (0.032) | -0.083**  (0.038) | -0.654***  (0.032) | -0.442***  (0.051) | -0.278***  (0.072) |
| Somalia | -0.390***  (0.076) | -0.391***  (0.068) | -0.351***  (0.075) | -0.453***  (0.044) | -0.361***  (0.056) | -0.326***  (0.059) |
| Syria | -0.380***  (0.103) | -0.251***  (0.083) | -0.303***  (0.086) | -0.269**  (0.110) | -0.230**  (0.095) | -0.164  (0.103) |
| Ethiopia | -0.044  (0.081) | -0.138*  (0.078) | -0.138  (0.084) | -0.010  (0.145) | -0.012  (0.114) | 0.049  (0.103) |
| Eritrea | -0.133  (0.124) | -0.010  (0.097) | -0.049  (0.092) | -0.072  (0.183) | 0.084  (0.117) | 0.189**  (0.091) |
| Bosnia | -0.180***  (0.028) | -0.131***  (0.022) | -0.103***  (0.028) | -0.164***  (0.023) | -0.019  (0.020) | 0.075**  (0.028) |
