# Supplementary material for: Refugee Employment Integration Heterogeneity in Sweden: Evidence From a Cohort Analysis
Source: Front Sociol. 2020 Jul 2;5:44. doi: 10.3389/fsoc.2020.00044 (PMC8022623; doi:10.3389/fsoc.2020.00044)
Supplement: Supplementary file 4 [file Table_4.DOCX]

Table 4. Regression Output Men (Detailed)

| Dep. Variable: Employed | Coef. | Std. Error | P-Value |
| --- | --- | --- | --- |
| *Country of Origin* |  |  |  |
| Iraq | -0.818 | 0.019 | 0.000 |
| Iran | -0.723 | 0.080 | 0.000 |
| Afghanistan | -0.778 | 0.074 | 0.000 |
| Somalia | -0.574 | 0.138 | 0.000 |
| Syria | -0.660 | 0.200 | 0.001 |
| Ethiopia | -0.363 | 0.169 | 0.032 |
| Eritrea | -0.528 | 0.215 | 0.014 |
| Bosnia | -0.780 | 0.054 | 0.000 |
| *Country of Origin x Years Since Migration* |  |  |  |
| Iraq | 0.163 | 0.008 | 0.000 |
| Iran | 0.089 | 0.026 | 0.001 |
| Afghanistan | 0.082 | 0.025 | 0.001 |
| Somalia | 0.089 | 0.050 | 0.075 |
| Syria | 0.087 | 0.059 | 0.137 |
| Ethiopia | 0.256 | 0.053 | 0.000 |
| Eritrea | 0.127 | 0.070 | 0.073 |
| Bosnia | 0.264 | 0.017 | 0.000 |
| *Country of Origin x Year Since Migration²* |  |  |  |
| Iraq | -0.011 | 0.002 | 0.000 |
| Iran | -0.002 | 0.004 | 0.673 |
| Afghanistan | 0.004 | 0.004 | 0.360 |
| Somalia | -0.011 | 0.009 | 0.180 |
| Syria | -0.003 | 0.010 | 0.731 |
| Ethiopia | -0.034 | 0.009 | 0.000 |
| Eritrea | -0.010 | 0.011 | 0.367 |
| Bosnia | -0.034 | 0.003 | 0.000 |
| *Country of Origin x Year Since Migration³* |  |  |  |
| Iraq | 0.000 | 0.000 | 0.000 |
| Iran | 0.000 | 0.000 | 0.700 |
| Afghanistan | 0.000 | 0.000 | 0.070 |
| Somalia | 0.001 | 0.000 | 0.198 |
| Syria | 0.000 | 0.000 | 0.862 |
| Ethiopia | 0.001 | 0.000 | 0.001 |
| Eritrea | 0.000 | 0.001 | 0.646 |
| Bosnia | 0.001 | 0.000 | 0.000 |
| Age | 0.003 | 0.001 | 0.000 |
| Age² | 0.000 | 0.000 | 0.001 |
| Age³ | 0.000 | 0.000 | 0.969 |
| *Country of Origin x Age* |  |  |  |
| Iraq | -0.027 | 0.004 | 0.000 |
| Iran | -0.019 | 0.018 | 0.285 |
| Afghanistan | -0.012 | 0.016 | 0.472 |
| Somalia | -0.001 | 0.030 | 0.979 |
| Syria | -0.001 | 0.036 | 0.980 |
| Ethiopia | -0.082 | 0.034 | 0.015 |
| Eritrea | 0.015 | 0.043 | 0.724 |
| Bosnia | -0.001 | 0.011 | 0.939 |
| *Country of Origin x Age²* |  |  |  |
| Iraq | 0.001 | 0.000 | 0.001 |
| Iran | 0.001 | 0.001 | 0.368 |
| Afghanistan | 0.000 | 0.001 | 0.888 |
| Somalia | -0.001 | 0.002 | 0.448 |
| Syria | 0.000 | 0.002 | 0.862 |
| Ethiopia | 0.005 | 0.002 | 0.026 |
| Eritrea | -0.002 | 0.003 | 0.442 |
| Bosnia | 0.000 | 0.001 | 0.970 |
| *Country of Origin x Age³* |  |  |  |
| Iraq | 0.000 | 0.000 | 0.000 |
| Iran | 0.000 | 0.000 | 0.310 |
| Afghanistan | 0.000 | 0.000 | 0.956 |
| Somalia | 0.000 | 0.000 | 0.353 |
| Syria | 0.000 | 0.000 | 0.803 |
| Ethiopia | 0.000 | 0.000 | 0.021 |
| Eritrea | 0.000 | 0.000 | 0.403 |
| Bosnia | 0.000 | 0.000 | 0.722 |
| *Education* |  |  |  |
| Primary education 9 years | 0.015 | 0.005 | 0.006 |
| Secondary education 2 years | 0.069 | 0.005 | 0.000 |
| Secondary education 3 years | 0.090 | 0.005 | 0.000 |
| University education 2 years | 0.096 | 0.005 | 0.000 |
| University education 3 years or more | 0.121 | 0.005 | 0.000 |
| PhD education | 0.129 | 0.006 | 0.000 |
| *Marital Status* |  |  |  |
| Single | -0.066 | 0.002 | 0.000 |
| Divorced | -0.031 | 0.010 | 0.002 |
| Widowed | -0.080 | 0.001 | 0.000 |
| Number of Children | 0.021 | 0.001 | 0.000 |
| *Year* |  |  |  |
| 1999 | 0.013 | 0.001 | 0.000 |
| 2000 | 0.022 | 0.001 | 0.000 |
| 2001 | 0.022 | 0.001 | 0.000 |
| 2002 | 0.025 | 0.001 | 0.000 |
| 2003 | 0.027 | 0.001 | 0.000 |
| 2004 | 0.026 | 0.002 | 0.000 |
| 2005 | 0.024 | 0.002 | 0.000 |
| 2006 | 0.033 | 0.002 | 0.000 |
| 2007 | 0.043 | 0.002 | 0.000 |
| 2008 | 0.041 | 0.002 | 0.000 |
| 2009 | 0.021 | 0.002 | 0.000 |
| 2010 | 0.009 | 0.014 | 0.521 |
| 2011 | 0.031 | 0.020 | 0.116 |
| 2012 | 0.012 | 0.027 | 0.649 |
| Stockholm | 0.017 | 0.002 | 0.000 |
| *Stockholm x Year* |  |  |  |
| 1999 | 0.001 | 0.650 | 0.005 |
| 2000 | 0.003 | 1.090 | 0.007 |
| 2001 | 0.003 | 1.380 | 0.008 |
| 2002 | -0.004 | -1.540 | 0.001 |
| 2003 | -0.015 | -5.490 | -0.009 |
| 2004 | -0.016 | -5.830 | -0.010 |
| 2005 | -0.019 | -6.830 | -0.013 |
| 2006 | -0.020 | -7.300 | -0.015 |
| 2007 | -0.020 | -7.480 | -0.015 |
| 2008 | -0.020 | -7.280 | -0.015 |
| 2009 | -0.013 | -4.590 | -0.007 |
| 2010 | 0.053 | 3.310 | 0.085 |
| 2011 | 0.027 | 1.340 | 0.067 |
| 2012 | 0.050 | 1.890 | 0.102 |
| Gothenburg | 0.004 | 0.003 | 0.100 |
| *Gothenburg x Year* |  |  |  |
| 1999 | 0.000 | -0.210 | 0.004 |
| 2000 | 0.002 | 0.720 | 0.007 |
| 2001 | 0.000 | 0.020 | 0.005 |
| 2002 | -0.001 | -0.440 | 0.004 |
| 2003 | -0.004 | -1.380 | 0.002 |
| 2004 | -0.004 | -1.390 | 0.002 |
| 2005 | -0.002 | -0.590 | 0.004 |
| 2006 | -0.005 | -1.900 | 0.000 |
| 2007 | -0.009 | -2.950 | -0.003 |
| 2008 | -0.007 | -2.320 | -0.001 |
| 2009 | -0.009 | -2.860 | -0.003 |
| 2010 | -0.027 | -1.380 | 0.011 |
| 2011 | -0.011 | -0.440 | 0.037 |
| 2012 | -0.017 | -0.540 | 0.046 |
| Malmö | -0.013 | 0.003 | 0.000 |
| *Malmö x Year* |  |  |  |
| 1999 | -0.001 | -0.440 | 0.004 |
| 2000 | -0.002 | -0.660 | 0.004 |
| 2001 | -0.002 | -0.540 | 0.004 |
| 2002 | -0.005 | -1.590 | 0.001 |
| 2003 | -0.010 | -2.970 | -0.003 |
| 2004 | -0.006 | -1.860 | 0.000 |
| 2005 | -0.002 | -0.640 | 0.004 |
| 2006 | -0.006 | -1.670 | 0.001 |
| 2007 | -0.008 | -2.480 | -0.002 |
| 2008 | -0.006 | -1.860 | 0.000 |
| 2009 | -0.007 | -1.950 | 0.000 |
| 2010 | -0.091 | -4.200 | -0.049 |
| 2011 | -0.118 | -4.330 | -0.065 |
| 2012 | -0.109 | -3.020 | -0.038 |
| Constant | 0.799 | 0.006 | 0.000 |
| R-Squared | 0.10 | | |
| Prob > F | 0.00 | | |
| Observations | 1,614,462 | | |
