# Supplementary material for: Refugee Employment Integration Heterogeneity in Sweden: Evidence From a Cohort Analysis
Source: Front Sociol. 2020 Jul 2;5:44. doi: 10.3389/fsoc.2020.00044 (PMC8022623; doi:10.3389/fsoc.2020.00044)
Supplement: Supplementary file 5 [file Table_5.DOCX]

Table 5. Regression Output Women (Detailed)

| Dep. Variable: Employed | Coef. | Std. Error | P-Value |
| --- | --- | --- | --- |
| *Country of Origin* |  |  |  |
| Iraq | -0.799 | 0.029 | 0.000 |
| Iran | -0.677 | 0.067 | 0.000 |
| Afghanistan | -0.770 | 0.072 | 0.000 |
| Somalia | -0.713 | 0.106 | 0.000 |
| Syria | -0.521 | 0.205 | 0.011 |
| Ethiopia | -0.490 | 0.272 | 0.071 |
| Eritrea | -0.321 | 0.372 | 0.387 |
| Bosnia-Hercegovina | -0.786 | 0.044 | 0.000 |
| *Country of Origin x Years Since Migration* |  |  |  |
| Iraq | 0.100 | 0.012 | 0.000 |
| Iran | 0.118 | 0.028 | 0.000 |
| Afghanistan | 0.026 | 0.036 | 0.476 |
| Somalia | 0.118 | 0.047 | 0.012 |
| Syria | 0.193 | 0.061 | 0.002 |
| Ethiopia | 0.290 | 0.068 | 0.000 |
| Eritrea | 0.129 | 0.097 | 0.184 |
| Bosnia-Hercegovina | 0.257 | 0.018 | 0.000 |
| *Country of Origin x Year Since Migration²* |  |  |  |
| Iraq | -0.007 | 0.002 | 0.002 |
| Iran | -0.006 | 0.005 | 0.209 |
| Afghanistan | 0.005 | 0.007 | 0.421 |
| Somalia | -0.010 | 0.008 | 0.237 |
| Syria | -0.018 | 0.011 | 0.107 |
| Ethiopia | -0.038 | 0.011 | 0.000 |
| Eritrea | -0.006 | 0.017 | 0.710 |
| Bosnia-Hercegovina | -0.028 | 0.003 | 0.000 |
| *Country of Origin x Year Since Migration³* |  |  |  |
| Iraq | 0.000 | 0.000 | 0.024 |
| Iran | 0.000 | 0.000 | 0.554 |
| Afghanistan | 0.000 | 0.000 | 0.370 |
| Somalia | 0.000 | 0.000 | 0.454 |
| Syria | 0.001 | 0.001 | 0.228 |
| Ethiopia | 0.002 | 0.001 | 0.002 |
| Eritrea | 0.000 | 0.001 | 0.859 |
| Bosnia-Hercegovina | 0.001 | 0.000 | 0.000 |
| Age | 0.009 | 0.001 | 0.000 |
| Age² | 0.000 | 0.000 | 0.482 |
| Age³ | 0.000 | 0.000 | 0.000 |
| *Country of Origin x Age* |  |  |  |
| Iraq | -0.018 | 0.007 | 0.006 |
| Iran | -0.037 | 0.016 | 0.020 |
| Afghanistan | -0.020 | 0.019 | 0.290 |
| Somalia | -0.023 | 0.023 | 0.309 |
| Syria | -0.083 | 0.035 | 0.019 |
| Ethiopia | -0.049 | 0.062 | 0.426 |
| Eritrea | -0.052 | 0.072 | 0.473 |
| Bosnia-Hercegovina | -0.007 | 0.010 | 0.486 |
| *Country of Origin x Age²* |  |  |  |
| Iraq | 0.001 | 0.000 | 0.020 |
| Iran | 0.002 | 0.001 | 0.029 |
| Afghanistan | 0.002 | 0.001 | 0.157 |
| Somalia | 0.001 | 0.002 | 0.590 |
| Syria | 0.004 | 0.002 | 0.063 |
| Ethiopia | 0.003 | 0.004 | 0.535 |
| Eritrea | 0.002 | 0.004 | 0.637 |
| Bosnia-Hercegovina | 0.000 | 0.001 | 0.636 |
| *Country of Origin x Age³* |  |  |  |
| Iraq | 0.000 | 0.000 | 0.002 |
| Iran | 0.000 | 0.000 | 0.009 |
| Afghanistan | 0.000 | 0.000 | 0.057 |
| Somalia | 0.000 | 0.000 | 0.570 |
| Syria | 0.000 | 0.000 | 0.098 |
| Ethiopia | 0.000 | 0.000 | 0.614 |
| Eritrea | 0.000 | 0.000 | 0.656 |
| Bosnia-Hercegovina | 0.000 | 0.000 | 0.176 |
| *Education* |  |  |  |
| Primary education 9 years | 0.051 | 0.009 | 0.000 |
| Secondary education 2 years | 0.176 | 0.008 | 0.000 |
| Secondary education 3 years | 0.210 | 0.008 | 0.000 |
| University education 2 years | 0.212 | 0.008 | 0.000 |
| University education 3 years or more | 0.269 | 0.008 | 0.000 |
| PhD ecucation | 0.270 | 0.009 | 0.000 |
| *Marital Status* |  |  |  |
| Single | -0.070 | 0.002 | 0.000 |
| Divorced | -0.062 | 0.008 | 0.000 |
| Widowed | -0.054 | 0.002 | 0.000 |
| Number of Children | 0.000 | 0.001 | 0.730 |
| *Year* |  |  |  |
| 1999 | 0.011 | 0.001 | 0.000 |
| 2000 | 0.011 | 0.002 | 0.000 |
| 2001 | 0.022 | 0.002 | 0.000 |
| 2002 | 0.021 | 0.002 | 0.000 |
| 2003 | 0.023 | 0.002 | 0.000 |
| 2004 | 0.015 | 0.002 | 0.000 |
| 2005 | 0.011 | 0.002 | 0.000 |
| 2006 | 0.017 | 0.002 | 0.000 |
| 2007 | 0.024 | 0.002 | 0.000 |
| 2008 | 0.026 | 0.002 | 0.000 |
| 2009 | 0.009 | 0.002 | 0.000 |
| 2010 | -0.002 | 0.019 | 0.926 |
| 2011 | -0.001 | 0.027 | 0.969 |
| 2012 | -0.041 | 0.037 | 0.271 |
| Stockholm | 0.043 | 0.003 | 0.000 |
| *Stockholm x Year* |  |  |  |
| 1999 | -0.003 | 0.002 | 0.201 |
| 2000 | -0.006 | 0.003 | 0.017 |
| 2001 | -0.016 | 0.003 | 0.000 |
| 2002 | -0.019 | 0.003 | 0.000 |
| 2003 | -0.021 | 0.003 | 0.000 |
| 2004 | -0.022 | 0.003 | 0.000 |
| 2005 | -0.019 | 0.003 | 0.000 |
| 2006 | -0.022 | 0.003 | 0.000 |
| 2007 | -0.024 | 0.003 | 0.000 |
| 2008 | -0.026 | 0.003 | 0.000 |
| 2009 | -0.021 | 0.003 | 0.000 |
| 2010 | -0.023 | 0.023 | 0.305 |
| 2011 | -0.037 | 0.029 | 0.193 |
| 2012 | -0.007 | 0.037 | 0.842 |
| Gothenburg | 0.013 | 0.003 | 0.000 |
| *Gothenburg x Year* |  |  |  |
| 1999 | 0.000 | 0.003 | 0.985 |
| 2000 | -0.007 | 0.003 | 0.018 |
| 2001 | -0.007 | 0.003 | 0.020 |
| 2002 | -0.008 | 0.003 | 0.008 |
| 2003 | -0.007 | 0.003 | 0.021 |
| 2004 | -0.010 | 0.003 | 0.004 |
| 2005 | -0.008 | 0.003 | 0.012 |
| 2006 | -0.009 | 0.003 | 0.006 |
| 2007 | -0.009 | 0.003 | 0.007 |
| 2008 | -0.008 | 0.003 | 0.013 |
| 2009 | -0.007 | 0.003 | 0.036 |
| 2010 | -0.037 | 0.026 | 0.151 |
| 2011 | -0.036 | 0.033 | 0.272 |
| 2012 | 0.012 | 0.042 | 0.775 |
| Malmö | -0.004 | 0.004 | 0.200 |
| *Malmö x Year* |  |  |  |
| 1999 | 0.000 | 0.003 | 0.933 |
| 2000 | -0.001 | 0.003 | 0.791 |
| 2001 | -0.004 | 0.004 | 0.309 |
| 2002 | -0.008 | 0.004 | 0.039 |
| 2003 | -0.005 | 0.004 | 0.216 |
| 2004 | -0.006 | 0.004 | 0.137 |
| 2005 | -0.004 | 0.004 | 0.280 |
| 2006 | 0.000 | 0.004 | 0.936 |
| 2007 | -0.002 | 0.004 | 0.655 |
| 2008 | -0.005 | 0.004 | 0.243 |
| 2009 | -0.004 | 0.004 | 0.326 |
| 2010 | -0.061 | 0.028 | 0.030 |
| 2011 | -0.105 | 0.037 | 0.004 |
| 2012 | -0.107 | 0.045 | 0.018 |
| Constant | 0.569 | 0.009 | 0.000 |
| R-Squared | 0.08 | | |
| Prob > F | 0.00 | | |
| Observations | 1,538,147 | | |
